# International risk of yellow fever spread from the ongoing outbreak in Brazil, December 2016 to May 2017

**DOI:** 10.2807/1560-7917.ES.2017.22.28.30572

**Published:** 2017-07-13

**Authors:** Ilaria Dorigatti, Arran Hamlet, Ricardo Aguas, Lorenzo Cattarino, Anne Cori, Christl A Donnelly, Tini Garske, Natsuko Imai, Neil M Ferguson

**Affiliations:** 1 MRC Centre for Outbreak analysis and Modelling, Department of Infectious Disease Epidemiology, Imperial College London, London, United Kingdom; 2 Centre for Tropical Medicine and Global Health, Nuffield Department of Medicine, University of Oxford, Oxford, United Kingdom; 3 Mahidol-Oxford Tropical Medicine Research Unit, Faculty of Tropical Medicine, Mahidol University, Bangkok, Thailand

**Keywords:** yellow fever, outbreak, Brazil, travel-related international spread

## Abstract

States in south-eastern Brazil were recently affected by the largest Yellow Fever (YF) outbreak seen in a decade in Latin America. Here we provide a quantitative assessment of the risk of travel-related international spread of YF indicating that the United States, Argentina, Uruguay, Spain, Italy and Germany may have received at least one travel-related YF case capable of seeding local transmission. Mitigating the risk of imported YF cases seeding local transmission requires heightened surveillance globally.

The south-east of Brazil was recently affected by the largest outbreak of Yellow Fever (YF) reported in a decade in Latin America, with 784 confirmed human cases and 267 confirmed deaths reported as of 31 May 2017 [1] (Figure, panels A-B). The outbreak has spread from Minas Gerais and Espírito Santo to São Paulo and Rio de Janeiro, thus raising public health concern about the establishment of urban transmission and the spread of YF beyond Brazil’s national border.

**Figure:**
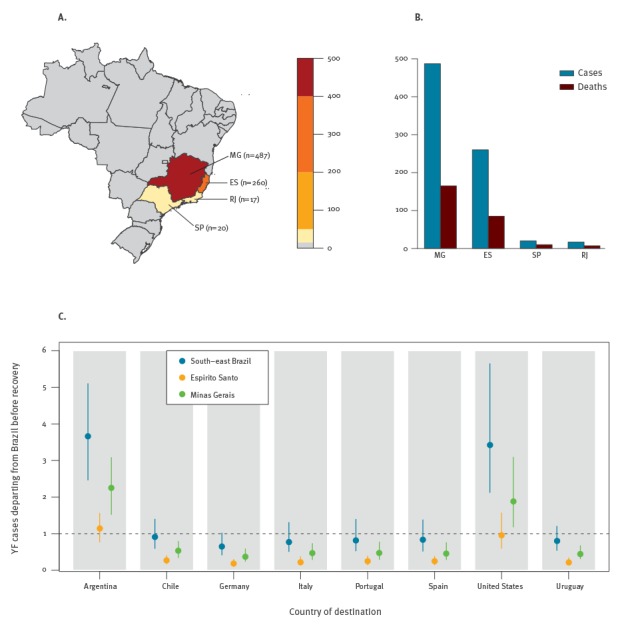
Confirmed yellow fever cases in south-east Brazil, 17 December 2016–31 May 2017 (n = 784)

ES: Espírito Santo; MG: Minas Gerais; RJ: Rio de Janeiro; SP: São Paulo; YF: yellow fever.

A. Geographical distribution of the range and cumulative number of confirmed cases reported by 31 May 2017 in the south-east of Brazil since December 2016 [1].

B. Cumulative number of confirmed cases and confirmed deaths by state reported as of 31 May 2017 [1].

C. Mean and 95% confidence interval of the estimated number of YF cases that could potentially seed a YF outbreak in the countries they are travelling to, comprising infected Brazilian residents travelling abroad during the incubation or infectious period (exportations) and international tourists infected by YF during their stay in the south-east of Brazil and returning to their home country (importations). The mean and 95% confidence intervals were obtained by numerically sampling 10,000 times the incubation and infectious period distributions [8,9]. Only destination countries with an upper 95% confidence limit exceeding one exported case over all states (south-east Brazil) are shown. The estimated risk of international spread from São Paulo and Rio de Janeiro is minimal and is not shown separately.

By linking the latest epidemiological data [1] with World Tourism Organisation data on the volume of air, land and water border crossings [2], we assessed the risk of travel-related international spread of YF.

## Data sources

The cumulative number of confirmed cases reported in the south-east of Brazil was obtained from the weekly epidemiological bulletins on YF published online by the Brazilian Ministry of Health [3]. The data used in this analysis refer to bulletin number 43 of 31 May 2017 [1].

For each state, the date of symptom onset of the first and last confirmed cases (Table) was retrieved from the time series reported in [1] using a web plot digitaliser tool [4].

**Table:** Date of symptom onset of first and last confirmed yellow fever cases reported per state, south-east Brazil, 17 December 2016–31 May 2017 (n = 784)

State	First date of symptom onset	Last date of symptom onset
Minas Gerais	19 Dec 2016	20 Apr 2017
Espírito Santo	4 Jan 2017	30 Apr 2017
São Paulo	17 Dec 2016	20 Apr 2017
Rio de Janeiro	19 Feb 2017	10 May 2017

Source: [1].

Population data for Brazil at country and state level relative to 2016 were obtained from the Brazilian Institute of Geography and Statistics website [5]. Data on the annual volumes of air, land and water border crossings for Brazil relative to inbound (arrivals of non-resident tourists at Brazilian national borders by country of residence) and outbound (trips abroad by Brazilian resident visitors to countries of destination) tourism for the year 2015 were purchased from the World Tourism Organisation (UNWTO) [2]. Information on the monthly distribution of inbound tourism and on the average duration of stay of international visitors to Brazil by country of origin was obtained from a survey on the touristic demand in Brazil conducted in 2015 [6].

## Modelling exportations and importations

We estimated the expected number of YF cases departing from Brazil during the incubation or infectious period, comprising infected residents of south-east Brazil travelling abroad (exportations) and international tourists infected by YF during their stay in the south-east of Brazil and returning to the home country (importations).

### Exportations

Let *C_S,W_* denote the cumulative number of confirmed YF cases reported in state *S* in time window *W*, with *W* denoting the number of days between the first and the last confirmed YF case in state *S*. Comparison of the observed case fatality ratio (CFR) [1] among confirmed cases (34.5%) and among confirmed and suspected cases (23%) in Brazil with the established CFR [7] among severe cases (47%; 95% confidence interval (CI): 31–62), mild or severe cases (13%; 95% CI: 5–28) and YF infections (comprising severe, mild and asymptomatic cases) (5%; 95% CI: 2–12) suggested that reported confirmed cases in the 2017 YF outbreak in Brazil are likely to be severe. Therefore, we assumed that all confirmed cases were severe and, following Johansson et al., that there were nine mild or asymptomatic infections for each severe case [7]. This implied that the cumulative number of YF cases in state *S* in time window *W* was given by ${\hat{C}}_{S,W}=10\cdot C_{S,W}$.

Let *pop_S_* denote the resident population of state S, *pop_B_* denote the resident population of the whole of Brazil and *T_D_* denote the annual number of Brazilian travellers visiting country *D*. The per capita probability that a Brazilian resident travelled to country *D* during time window *W* was given by $p_{D}=\frac{T_{D}\cdot W}{365}\cdot \frac{1}{pop_{B}}$.

We assumed that the incubation period *T_E_* was log-normally distributed with mean 4.6 days and variance 2.7 days [8] and that the infectious period *T_I_* was normally distributed with mean 4.5 days and variance 0.6 days [9]. The probability *p_i_* that a YF case incubated or was infectious in time window *W* was$p_{i}=\frac{T_{E}+T_{I}}{W}$.

The number of residents of state *S* infected by YF virus and travelling abroad during their incubation or infectious period in time window *W* was given by $E_{S,W}={\hat{C}}_{S,W}\cdot p_{D}\cdot p_{i}$.

### Importations

Let *T_O_* denote the annual number of travellers visiting Brazil from country *O*, *f_m_* the proportion of international travellers visiting Brazil in month *m* and *p_s,m_* the relative proportion of the epidemic window *W* in state *S* occurring in month *m*. Assuming that travellers to Brazil pick destination states within the country with a probability proportional to the states’ population sizes, the expected number of travellers $T_{O,S}^{W}$ visiting state *S* from country *O* in in time window *W* was given by $T_{O,S}^{W}=T_{O}\left(\sum_{m=1}^{12}f_{m}p_{S,m}\right)\cdot \frac{pop_{S}}{pop_{B}}$.

Let *L_O_* denote the average length of stay of travellers visiting Brazil from country *O*. The per capita risk of infection of travellers visiting state S during their stay was estimated as $\lambda_{S}=\frac{{\hat{C}}_{S,W}\cdot L_{O}}{pop_{S}\cdot W}$.

The probability of returning to the home country while incubating or infectious was $p_{l}=\frac{T_{E}+T_{I}}{L_{O}}$, where *p_l_* was set to 1 if *(T_E_ + T_I_) > L_O_* .

The expected number of international tourists infected by YF during their stay in state *S* and returning to the home country *O* before the end of the infectious period was estimated by $I_{S,O}=T_{O,S}^{W}\cdot \lambda_{S}\cdot p_{l}$.

Variability in the incubation and infectious periods was accounted for by sampling 10,000 times *T_E_* and *T_I_* from their respective distributions, leading to a full distribution for *p_l_* and in turn for *E_S,W_* and *I_S,O_*.

## International risk of travel-related yellow fever spread 

We show in the Figure (panel C) the expected number of YF cases departing from Brazil before recovery (i.e. during the incubation or infectious period), comprising exportations and importations, for the destination countries with an upper 95% confidence limit exceeding one case over all states in the south-east of Brazil. We found that the United States, Latin America (specifically Argentina, Chile and Uruguay), and Europe (specifically Germany, Italy, Portugal and Spain) may have already received at least one travel-related YF case capable of seeding local transmission. Sensitivity analysis showed that the expected number of YF cases departing from Brazil before recovery was robust to alternative assumptions on the distribution of international travellers across the Brazilian states (e.g. according to rural/urban indicators) and that exportations were the biggest source of travel-related spread of YF. 

## Discussion 

The southern United States, Argentina and Uruguay contain regions where *Aedes aegypti* mosquitos, the most competent vector species for YF transmission, are established [10]. While *Aedes aegypti* mosquitoes are not present in Europe, except on Madeira, *Ae. albopictus* mosquitoes, which are potentially also competent to transmit the YF virus, have been reported in Germany, and established populations have been observed in Spain, Italy [11] and in the south and north-east of the United States [10]. To date, however, there has been no evidence of natural YF transmission by *Ae. albopictus* in any part of the world. In continental Portugal and Chile, the presence of competent YF vectors has not been documented [10-12], although both countries are considered climatically and environmentally suitable [13-15]. With no new YF cases reported in Brazil since 31 May 2017, the 2017 YF outbreak in Brazil currently appears under control. We estimated that international travel-related YF spread may have occurred during the outbreak, implying that increased awareness, monitoring and preparedness was therefore appropriate to avoid the current YF outbreak in Brazil seeding new YF outbreaks globally. 
